# Same, same but different: symbiotic bacterial associations in GBR sponges

**DOI:** 10.3389/fmicb.2012.00444

**Published:** 2013-01-18

**Authors:** N. S. Webster, H. M. Luter, R. M. Soo, E. S. Botté, R. L. Simister, D. Abdo, S. Whalan

**Affiliations:** ^1^Australian Institute of Marine ScienceTownsville, QLD, Australia; ^2^Northern Australian Marine Research Alliance, Arafura Timor Research Facility DarwinBrinkin, NT, Australia; ^3^Research Institute for the Environment and Livelihoods, Charles Darwin UniversityCasuarina, NT, Australia; ^4^Australian Centre for Ecogenomics, University of QueenslandBrisbane, QLD, Australia; ^5^Centre for Microbial Innovation, School of Biological Sciences, The University of AucklandAuckland, New Zealand; ^6^Department of Fisheries, Government of Western AustraliaNorth Beach, WA, Australia; ^7^Marine Ecology Research Centre, School of Environment, Science and Engineering, Southern Cross UniversityLismore, NSW, Australia

**Keywords:** sponge, microorganism, symbiont, diversity, Great Barrier Reef

## Abstract

Symbioses in marine sponges involve diverse consortia of microorganisms that contribute to the health and ecology of their hosts. The microbial communities of 13 taxonomically diverse Great Barrier Reef (GBR) sponge species were assessed by DGGE and 16S rRNA gene sequencing to determine intra and inter species variation in bacterial symbiont composition. Microbial profiling revealed communities that were largely conserved within different individuals of each species with intra species similarity ranging from 65–100%. 16S rRNA gene sequencing revealed that the communities were dominated by *Proteobacteria*, *Chloroflexi, Acidobacteria, Actinobacteria, Nitrospira*, and *Cyanobacteria*. Sponge-associated microbes were also highly host-specific with no operational taxonomic units (OTUs) common to all species and the most ubiquitous OTU found in only 5 of the 13 sponge species. In total, 91% of the OTUs were restricted to a single sponge species. However, GBR sponge microbes were more closely related to other sponge-derived bacteria than they were to environmental communities with sequences falling within 50 of the 173 previously defined sponge-(or sponge-coral) specific sequence clusters (SC). These SC spanned the *Acidobacteria, Actinobacteria, Proteobacteria, Bacteroidetes, Chloroflexi, Cyanobacteria, Gemmatimonadetes, Nitrospira*, and the *Planctomycetes-Verrucomicrobia-Chlamydiae* superphylum. The number of sequences assigned to these sponge-specific clusters across all species ranged from 0 to 92%. No relationship between host phylogeny and symbiont communities were observed across the different sponge orders, although the highest level of similarity was detected in two closely related *Xestospongia* species. This study identifies the core microbial inhabitants in a range of GBR sponges thereby providing the basis for future studies on sponge symbiotic function and research aiming to predict how sponge holobionts will respond to environmental perturbation.

## Introduction

Associations between sponges and bacteria have existed for 600 million years making them one of the most ancient of all symbioses between microbes and metazoa (Wilkinson, 1984). Most sponges host diverse and abundant communities of microorganisms (Hentschel et al., 2006; Taylor et al., 2007; Webster et al., 2010), which contribute to host health, ecology and evolution [reviewed in (Taylor et al., 2007) and (Webster and Taylor, 2012)]. The importance of the relationship between sponges and their associated microbial communities is supported by the fact that microorganisms can contribute to more than 35% of the sponge biomass (Vacelet, 1975) and undertake diverse functional roles including nutrition, cycling of metabolites and host defense [reviewed in (Hentschel et al., 2012; Webster and Taylor, 2012)]. At least 32 bacterial phyla and candidate phyla have so far been found in sponges, either via cultivation or molecular characterization (Taylor et al., 2007; Schmitt et al., 2012b; Webster and Taylor, 2012). Some of these may be transient members of the sponge microbiota, potentially being filtered from the seawater as food. However, the “core” taxa, thought to represent the stable sponge inhabitants or true symbionts, include the *Acidobacteria, Actinobacteria, Chloroflexi, Cyanobacteria, Gemmatimonadetes, Nitrospira, Proteobacteria*, (especially *Alpha*, *Delta*, *Gamma* classes) and the candidate phylum “*Poribacteria*” (Taylor et al., 2007; Schmitt et al., 2012b).

The existence of sponge-specific microorganisms was first reported over a decade ago based on the finding that distantly related sponges from geographically separated regions shared microbes that had not been recovered from any other source, including the surrounding seawater (Hentschel et al., 2002; Taylor et al., 2007). Clusters of sponge-specific sequences were defined if groups of at least three rRNA gene sequences derived from more than two sponge species shared higher similarity to each other compared to sequences from other environments (Hentschel et al., 2002). Recently, the concept of sponge-specific microbes was comprehensively explored by performing phylogenetic analyses of all publicly available 16S and 18S rRNA gene sequences that originated from sponges. In total, 27% of all sponge-derived sequences fell into monophyletic, sponge-specific sequence clusters (SC) within the Bacteria, Archaea, and Fungi (Simister et al., 2012a) and additional sequences fell within clusters containing both sponge and coral derived sequences (SCC). Within the Bacteria, *Chloroflexi, Cyanobacteria*, “*Poribacteria*”, *Betaproteobacteria*, and *Acidobacteria* contained the highest abundance of these SCs. However, deep sequencing of diverse marine environments including seawater, sediments, hydrothermal vents, salt marshes, microbial mats, and corals has recently demonstrated that the rare biosphere may be a reservoir for some previously designated “sponge-specific” microbes (Webster et al., 2010; Taylor et al., 2012).

It is well established that particular sponge species can host stable microbial populations that are different to the communities in other species (Wilkinson et al., 1981; Taylor et al., 2004, 2005; Webster et al., 2004, 2010; Holmes and Blanch, 2007; Erwin and Thacker, 2008b; Turque et al., 2008; Lee et al., 2009; Erwin et al., 2011, 2012a,b; Schmitt et al., 2012b) and some molecular evidence also supports the potential for host-symbiont coevolution (Erpenbeck et al., 2002; Thacker and Starnes, 2003; Fan et al., 2012). Throughout the sponge literature and within this manuscript, the term “symbiosis” is used in its loosest possible definition, referring to the stable host-microbe association rather than implying any symbiotic function to the relationship. To test whether geographic or host-specific subpopulations of sponge microbes exist, Schmitt and colleagues performed 454 amplicon sequencing on 32 sponge species collected from eight locations around the world (Schmitt et al., 2012b). Whilst tropical sponge species shared more similarity in their microbial communities than they did with sub-tropical species, no other geographic or host phylogeny patterns were detected (Schmitt et al., 2012b). Interestingly, only a small “core” bacterial community was present in all 32 sponge species with the majority of bacterial operational taxonomic units (OTUs) occurring in only a single sponge species. Whilst the different sponge species were found to contain unique microbial populations, most of these sponge-associated bacteria were more closely related to other sponge-derived bacteria than they were to microbes from other environmental origins. These findings suggest that exploration of further sponge species may reveal additional novel sequences which would enhance our understanding of these sponge-specific microbial communities.

Over 8500 sponge species have been described globally (van Soest et al., 2012) with an estimated 2500 species occurring in Australian waters, although many of these remain to be formally described (Hooper, 2009). Baseline data on the composition and stability of symbiotic microbial communities is lacking for most sponge species and this knowledge gap makes it difficult to determine the role of microorganisms in sponge morbidity and mortality events. Sponge disease and mass mortalities have increased over recent years (Webster, 2007; Maldonado et al., 2010; Angermeier et al., 2011, 2012), including numerous reports of diseases that affect Great Barrier Reef (GBR) sponge species (Webster et al., 2002; Webster, 2007; Luter et al., 2010a,b). The study of sponge disease (including the identification of causative agents) is hampered by the high bacterial diversity within sponges combined with inadequate knowledge of the inherent microbial communities for most species. Understanding how sponges are likely to respond to a rapidly changing environment has also been a recent research focus (Webster et al., 2008; López-Legentil et al., 2010; Simister et al., 2012b), but scientists require a much better understanding of the diversity and specificity of microbes before assessments of environmental change can be validated.

Enhanced understanding of the ecological and evolutionary implications of sponge-bacterial symbioses gained over the past decade has prompted considerable new research in this field (Webster and Taylor, 2012), however, some regions such as the GBR remain largely understudied. The GBR is home to over 1500 sponge species although the microbial associates of the vast majority of species are yet to be explored. Here we surveyed the taxonomic composition of microbial associates in 13 of the most abundant and ecologically important GBR sponge species spanning five orders within the Porifera. By profiling replicate individuals per sponge species we are also able to address questions related to intra and inter species specificity.

## Materials and methods

### Sample collection

Sponge species were selected to encompass a wide range of genera covering five taxonomic orders: (1) Dictyoceratida included *Luffariella variabilis, Coscinoderma matthewsi, Carteriospongia foliascens*, and *Ircinia* sp. (2) Haplosclerida included *Xestospongia testudinaria, Xestospongia exigua*, and *Haliclona* sp. (3) Poescilosclerida included *Coelocarteria singaporensis, Paramyxilla* sp., and *Hamigera* sp. (4) Halichondrida included *Stylissa* sp. and *Cymbastella coralliophila* and (5) Spirophorida included *Cinachyra* sp. Triplicate individuals per species were collected on SCUBA from Orpheus Island (18°33.617′S; 146°29.077′E) at a depth range of 5–10 m in July 2010. Samples were photographed, immediately frozen in liquid nitrogen and stored at −80°C until subsequent molecular analysis.

### DNA extraction, DGGE and 16S rRNA gene sequence analysis

DNA was extracted from all sponge samples using the Power Plant DNA Isolation kit, MoBio Laboratories (Carlsbad, CA) according to the manufacturer's protocol. The 16S rRNA gene from each sponge sample was amplified by PCR with primers 1055f and 1406r (Ferris et al., 1996) with the reverse primer incorporating the GC clamp (Muyzer et al., 1993). PCR reactions were performed as described by Ferris et al. (1996). Products from triplicate PCR reactions were combined and 15 μl applied to duplicate 8% w/v polyacrylamide (37.5:1) gels containing a 50–70% denaturing gradient of formamide and urea. Gels were electrophoresed at 60°C for 17 h in 1 × TAE buffer at 50 V using the Ingeny D-Code system. Gels were stained with 1 × Sybr Gold for 30 min, visualized under UV illumination and photographed. Representative bands from each species were excised and sequenced using the forward primer at Macrogen Inc., the PRISM Ready Reaction Kit (PE Applied Biosystems) and the ABI 310 and 373 automated sequencers.

For a more comprehensive phylogenetic comparison between the different sponge species, the 16S rRNA gene from triplicate samples of each sponge species was amplified by PCR with primers 63f and 1387r (Marchesi et al., 1998). PCR products were combined for all sponge replicates per species and ligated into the TOPO TA cloning vector (Invitrogen). Ligations were sent to Macrogen Inc for transformation and sequencing of up to 96 clones for each library. All sequences were submitted to Genbank under accession numbers JX455220—JX455743.

### Data analysis (DGGE)

16S rRNA gene fingerprints of the DGGE gels were manually assessed and a matrix was constructed using the presence (1) or absence (0) of a band in each sample. All analyses of the DGGE data were conducted using the Primer + PERMANOVA software package.

To determine if the microbial communities were significantly different between host species, Permutational Multivariate Analysis of Variance (PERMANOVA) was conducted using a Euclidean distance matrix and 9999 permutations (Anderson et al., 2008). The analysis consisted of one fixed factor (host species) for all samples. PERMANOVA pair-wise comparisons were subsequently made amongst the levels of each significant factor (using the Euclidean distance matrix and 9999 permutations).

Canonical analysis of principal coordinates (CAP) was then used to display differences in the microbial community structure among the different sponge species (Anderson et al., 2008) and DGGE bands with a correlation greater than 0.5 were overlaid on the plots as vectors. Cluster analysis was performed using Group Average and Bray–Curtis similarity.

### Data analysis (16S rRNA gene sequences)

Clone sequence quality was checked manually using Sequencher (Genesearch, Brisbane). Chimeric sequences were identified using the programs CHECK_CHIMERA (Maidak et al., 1999) and Bellerophon (Huber et al., 2004). Sequences were imported into the ARB software package (http://www.arb-home.de) (Ludwig et al., 1998), automatically aligned using WebAligner and manually edited. Phylogenetic trees were calculated with almost complete 16S rRNA (1400 bp) sequences for all close relatives of target sequences using the neighbor-joining and Maximum Parsimony methods in ARB. Partial sequences were subsequently imported to the tree without changing branch topology using the ARB parsimony-interactive method. Taxonomic bar charts were constructed using the phylogenetic assignments produced in ARB and plotted using Sigmaplot (V7.101, SPSS Inc.).

Distance matrices were generated in MOTHUR V1.8.1 (DeSantis et al., 2006) using a Jukes Cantor correction and OTU assignment, diversity estimates and OTU heatmaps were subsequently produced in MOTHUR using a distance of 0.03 (Schloss et al., 2009). All published, sponge-derived sequences within a manually curated SILVA reference database were used to assign clone sequences to previously defined sponge-specific and sponge/coral-specific SC (Simister et al., 2012a). In brief, a BLAST search against the curated SILVA database was performed for each clone sequence and the 10 best hits were aligned so that sequence similarity could be determined. A 75% sequence similarity threshold was applied to assign the clone sequences to SC or SCCs.

## Results

**Figure 1:**
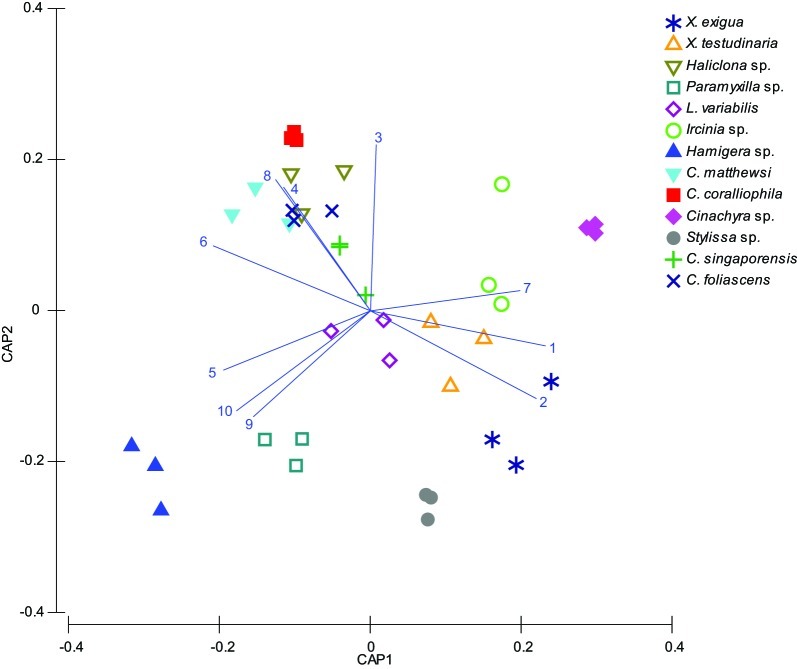
**CAP Analysis of microbial communities (based on DGGE banding pattern data) for different sponge species which were all sampled in triplicate (depicted by each shape).** DGGE bands with a correlation greater than 0.5 have been overlaid on the plots as vectors and the sequence identity of these are reported in Table 2.

**Figure 2:**
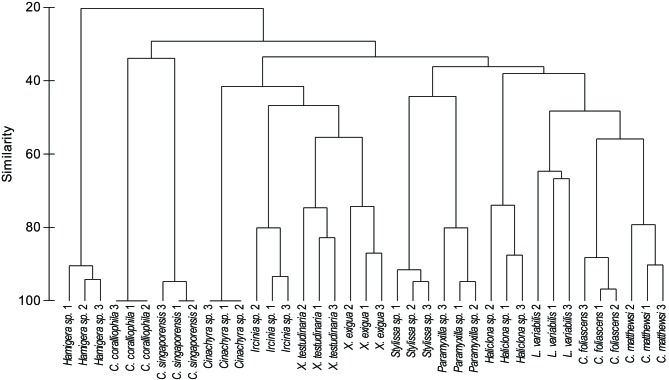
**Cluster analysis (based on DGGE banding pattern data) for different sponge species using Group Average and Bray Curtis similarity**.

A microbial profiling approach revealed that GBR sponge-associated microbial communities are highly conserved within each species (Figures 1, 2), but differ significantly between host species (*p* = 0.0001, Table 1, Figures 1, 2). Ordination analysis of the DGGE data showed clear clustering of replicate samples for all GBR sponge species (Figure 1). In particular, microbial communities in triplicate individuals of *C. coralliophila*, and *Cinachyra* sp. were identical within each species as were two of the three *C. singaporensis* replicates (Figure 2). *L. variabilis* had the greatest intra-species variation but individuals still shared >65% similarity in microbial community composition (Figure 2). Microorganisms that contributed most to the discrimination (correlation greater than 0.5, Figure 1) between host sponges were affiliated with the Archaea and the bacterial phyla *Proteobacteria*, *Chloroflexi* and *Cyanobacteria* with BLAST analysis showing that most sequences had highest similarity to microbes previously retrieved from sponges and corals (Table 2).

**Table 1:** **Summary PERMANOVA statistics (as per Anderson et al., 2008) of the DGGE derived symbiont assemblages reveal that microbial communities differ significantly between host sponge species (*p* = 0.0001)**.

**Source**	**df**	**SS**	**MS**	**Pseudo-F**	***P*(perm)**	**Unique perms**
Sponge	12	81497	6791.4	44.902	0.0001	9857
Residual	26	3932.5	151.25			
Total	38	85430				

*df, degrees of freedom; SS, sum of squares; MS, mean square; Unique perms, number of unique permutations*.

**Table 2:** **Microbial sequences detected by DGGE that discriminate between sponge hosts (correlation >0.5)**.

**Vector**	**Closest blast match**	***S***
1	*Deltaproteobacteria* from the coral *Montastrea faveolata* (GU118564)	99
2	*Chloroflexi* from the sponge *Xestospongia testudinaria* (JN596651)	99
3	*Gammaproteobacteria* from the hydra *Hydra magnipapillata* (FJ517701)	95
4	*Synechococcus spongiarum* from the sponge *Aplysina lacunose* (EU307503)	99
5	*Cenarchaeaceae* from the sponge *Axinella verrucosa* (AF420237)	99
6	*Thaumarchaea* from ALOHA seawater station (EF106806)	92
7	*Gammaproteobacteria* from Chinese soil	95
8	*Chloroflexi* associated with the sponge *Xestospongia muta* (FJ481375)	98
9	*Crenarchaea* from the marine environment (AF151345)	90
10	Unidentified bacterium associated with an Antarctic sponge (AY320217)	97

*Sequence identification determined using the most similar BLAST match from the NCBI database and S indicates % similarity*.

Pairwise comparisons revealed interesting patterns in the microbial assemblages amongst the sampled sponges (Table 3). For example, *L. variabilis* had a “generalist” microbial community that did not differ significantly from many of the other sampled sponges, whereas the microbial communities in *Hamigera* sp., *Cinachyra* sp., *C. coralliophila*, and *C. singaporensis* were significantly different to most other sponge species (Table 3). Cluster analysis confirmed that the microbial community in *Hamigera* sp. shared only 20% similarity to the other species (Figure 2). The highest similarity detected between host species (55%) occurred in the two *Xestospongia* species (Figure 2). Despite the similarity between *X. testudinaria* and *X. exigua*, no discernable clustering according to higher host taxonomy was evident. Three of the four Dictyoceratid species (*L. variabilis, C. matthewsi*, and *C. foliascens*) grouped together but this grouping shared less than 50% similarity in their microbial communities (Figure 2).

**Table 3:**
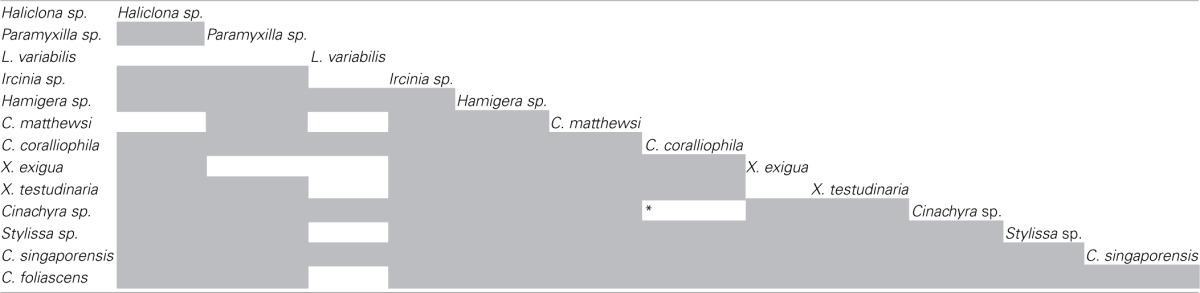
**Significant PERMANOVA pairwise comparisons of the microbial communities amongst the different sponge species**.

Due to the low number of permutations, Monte Carlo P-values were calculated and are also used to determine significant differences amongst the comparisons indicated by shaded cells (at P < 0.0043, Bonferroni corrected) ^*^indicates that no test was performed as the denominator was zero.

**Figure 3:**
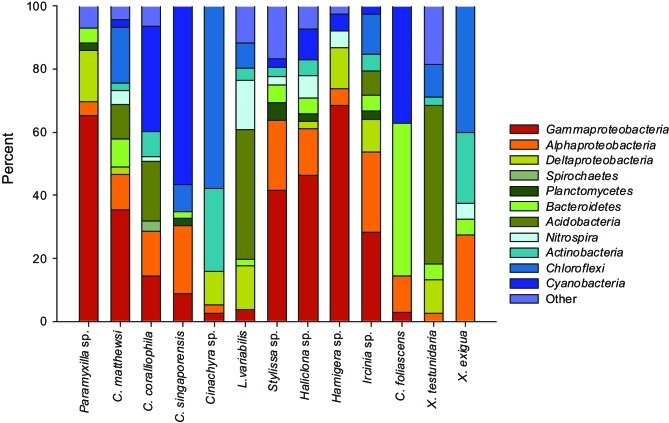
**Bar charts showing the relative abundance of each bacterial phyla (and class for the *Proteobacteria*) within each host species**.

16S rRNA gene libraries revealed diverse bacterial assemblages with clear taxonomic differences in the microbial communities inhabiting each sponge species (Figure 3). *Paramyxilla* sp., *C. matthewsi, Stylissa* sp., *Haliclona* sp., and *Hamigera* sp. were dominated by *Gammaproteobacteria*; *Cinachyra* sp. and *X. exigua* were dominated by *Chloroflexi* with a large proportion of *Actinobacteria; L. variabilis* and *X. testudinaria* were dominated by *Acidobacteria* and *C. coralliophila, C. singaporensis*, and *C. foliascens* contained a large abundance of *Cyanobacteria* (although *C. foliascens* was actually dominated by *Bacteroidetes*). *Ircinia* sp. had a more diverse and even distribution of bacterial phyla and classes within the *Proteobacteria* (Figure 3). No patterns according to host taxonomy were observed in the microbial community composition.

**Figure 4:**
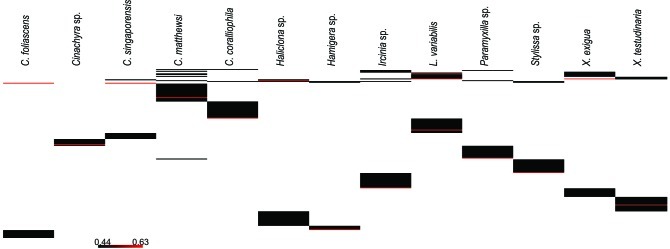
**OTU heatmaps generated with a distance of 0.03 for clone libraries from each sponge species.** Whilst there are a small number of shared OTUs, the vast majority are species-specific.

Whilst it was not possible to accurately estimate total sponge microbial diversity due to the low sequencing depth, comparison of diversity estimates within the GBR sponges (Table 4) indicated that *X. testudinaria, C. matthewsi* and *L. variabilis* hosted higher bacterial diversity, whereas *Cinachyra* sp., *C. foliascens*, *Hamigera* sp., and *C. singaporensis* had lower microbial diversity. Whilst two of the three species with highest bacterial diversity (*C. matthewsi* and *L. variabilis*) resided within the Dictyoceratida, the low microbial diversity species encompassed three different taxonomic orders. No observed OTUs (97% sequence similarity) were common to all sponge species (Figure 4) and the most ubiquitous OTU was shared between only five sponge species (*Stylissa* sp., *Haliclona* sp., *Ircinia* sp., *C. coraliophilla*, and *Hamigera* sp.). In total, 91% of the observed OTUs were detected in a single sponge species, 6% occurred across two species, 2% across three species and only 1% occurred in four or more species. These results indicate that the vast majority of dominant microbes (i.e., those detected at low sequencing depth) are species-specific (Figure 4).

**Table 4:** **Diversity indices calculated from sequences of 16S rRNA genes using a 97% sequence similarity threshold**.

**Sponge species**	**Total clones**	**Unique OTUs**	**%Reads SC/SCC**	**Chao 1**	**Ace**	**Shannon weaver**
*C. matthewsii*	37	28	54	58	73	3.2
*Haliclona* sp.	36	20	10	46	41	2.8
*Hamigera* sp.	24	7	0	7	9	1.6
*L. variabilis*	50	25	92	70	95	2.9
*C. singaporensis*	40	9	63	19	16	1.4
*Paramyxilla* sp.	41	17	2	31	36	2.2
*Stylissa* sp.	39	18	38	44	52	2.3
*C. foliacsens*	31	9	81	17	30	1.6
*Ircinia* sp.	34	23	68	57	65	3.0
*Cinachyra* sp.	36	8	69	11	14	1.3
*X. exigua*	37	17	78	20	33	2.5
*X. testudinaria*	33	20	85	140	126	2.7
*C. concentrica*	56	22	25	31	58	2.6

*SC / SCC refer to what percentage of sequences fell within previously defined “sponge-specific” or “sponge/coral-specific” sequence clusters. Chao 1, Ace and shannon weaver are diversity estimates calculated in Mothur*.

**Figure 5:**
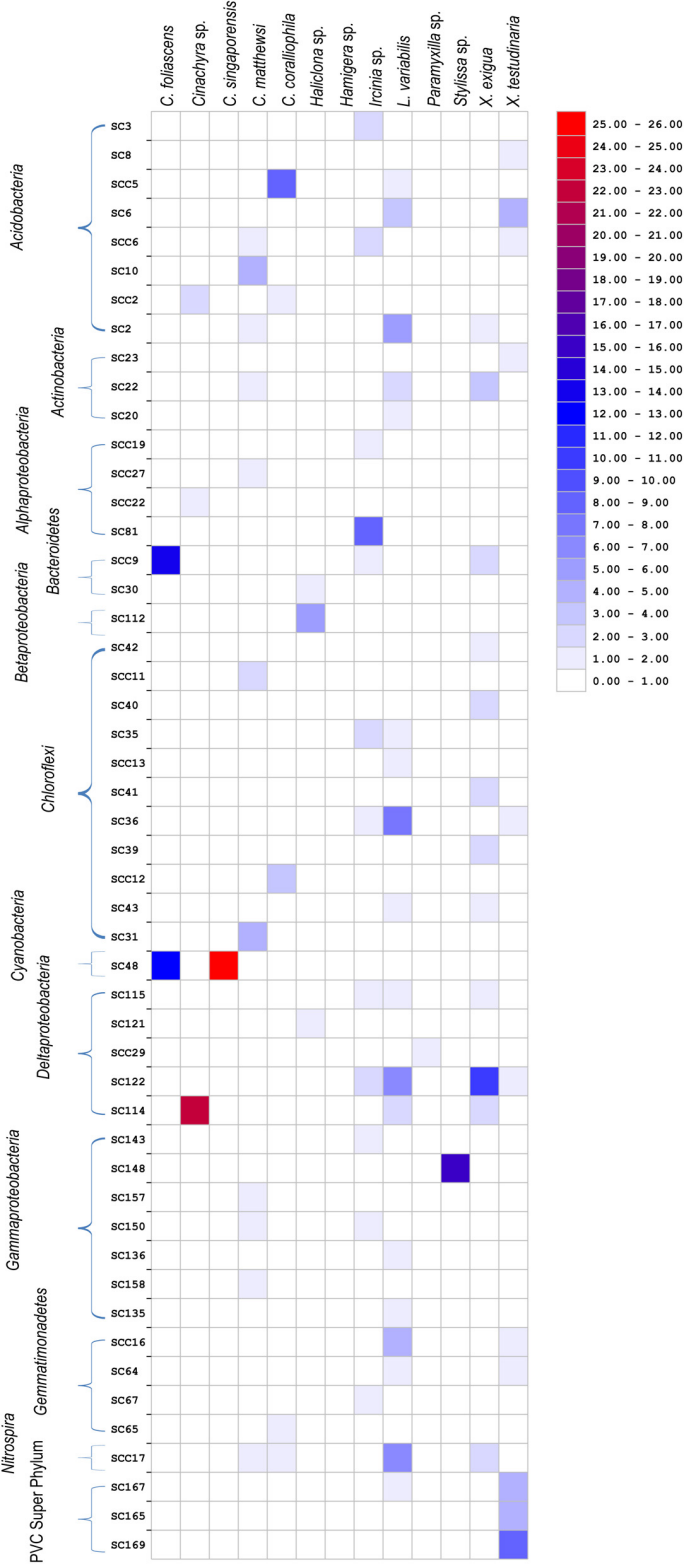
**Heatmap showing the distribution of clone sequences phylogenetically assigned to previously described “sponge-specific” 16S rRNA gene sequence clusters (Simister et al., 2012a) among the 13 different sponge species.** Clusters with an SC prefix contain sequences previously reported only from sponges; SCC prefix signifies clusters containing only sponge- and coral-derived sequences. Units are the percentage of total sequences from each sponge species that fall within SC/SCC.

Bacterial sequences retrieved from the 13 GBR sponge species fell within 50 of the 173 previously defined SC and SCCs. These SC and SCCs encompassed nine phyla including *Acidobacteria, Actinobacteria, Proteobacteria, Bacteroidetes, Chloroflexi, Cyanobacteria, Gemmatimonadetes, Nitrospira*, and the *Planctomycetes-Verrucomicrobia-Chlamydiae* superphylum (Figure 5). The number of sequences falling within SC/SCCs varied greatly between sponges, ranging from 0% in *Hamigera* sp. to 92% of the total sequences in *L. variabilis* (Table 4). No SC/SCCs were represented in more than 4 of the 13 sponge species with the most ubiquitous clusters being SC122 (*Deltaproteobacteria*) and SCC 17 (*Nitrospira*) which occurred in four different sponge species (Figure 5).

For the five species dominated by *Gammaproteobacteria*; *Paramyxilla* sp.,* Haliclona* sp. and *Hamigera* sp. contained no sequences residing within *Gammaproteobacteria* SC/SCCs whereas the *Gammaproteobacteria* in *C. matthewsi* were spread across three different SCs and all of the *Gammaproteobacteria* within *Sylissa* sp. clustered within a single SC. Whilst *C. foliascens, C. singaporensis* and *C. coralliophila* all contained abundant *Cyanobacteria*, only the first two species contained sequences within *Cyanobacteria* SC. Both *Xestospongia* species contained abundant sequences within SC/SCCs from multiple bacterial phyla. As illustrated in Figure 3, *C. matthewsi, Ircinia* sp. and *L. variabilis* had diverse bacterial assemblages and this was also evident by the relatively broad spread of SC/SCCs across taxa (Figure 5).

## Discussion

The microbial communities of taxonomically diverse GBR sponges were dominated by phyla previously reported to associate with marine sponges. The microbial populations were highly conserved within different individuals of each species yet varied significantly between species. This is consistent with patterns for other GBR sponges (Luter et al., 2010b, 2012; Webster et al., 2011; Fan et al., 2012) and in species from other broad geographic locations (Schmitt et al., 2012b; Webster and Taylor, 2012). Whilst BLAST analysis did not reveal common indicator species between the DGGE and the clone library analyses, both datasets were dominated by sequences that had sponge and coral derived bacteria as their closest relatives. Further highlighting the specific nature of GBR sponge-bacterial populations was the finding that all species (with the exception of *Hamigera* sp.) contained clone sequences that fell within previously defined clusters of sponge-specific bacterial sequences (Simister et al., 2012a).

The dominance of *Proteobacteria, Chloroflexi, Acidobacteria, Actinobacteria, Nitrospira*, and *Cyanobacteria* across the GBR sponge species accords with the dominant microbial groups described from other studies of sponge-associated microbial assemblages (Taylor et al., 2007; Webster and Taylor, 2012). Two of the GBR sponge species from this study (*C. coralliophila* and *Stylissa* sp.) were recently assessed using metagenomic shotgun sequencing (Fan et al., 2012). Whilst the different sequencing approaches were generally consistent, some methodological bias was evident with metagenomic sequencing detecting an abundance of *Chloroflexi* in *C. coralliophila* and a dominance of *Archaea* in *Stylissa* sp. which were not detected by DGGE or clone sequencing. The community composition in *X. testudinaria* from the present study showed high similarity with the microbial community described for this species in Indonesia (Montalvo and Hill, 2011). However, *Cyanobacteria*, which are well studied associates of Caribbean *Xestospongia* spp., were not detected in either of the GBR *Xestospongia* species (Steindler et al., 2005; Erwin and Thacker, 2007, 2008a). Episodes of both cyclic and fatal bleaching have dramatically effected populations of *Xestospongia muta* in the Caribbean (López-Legentil et al., 2008; McMurray et al., 2011), whereas there is currently no evidence of bleaching within GBR *Xestospongia* species. The absence of photosynthetic cyanobacteria in GBR species not only distinguishes these geographically separated congeners, but raises questions of GBR populations being less vulnerable to bleaching impacts associated with a changing climate. Results from the present study indicate that *C. singaporensis*, *C. foliascens*, and *C. coralliophila* are likely to be phototrophic species due to the high representation of *Cyanobacteria*. This is an important finding for studies that may want to distinguish differential responses of heterotrophs or phototrophs to environmental perturbation.

The *Chloroflexi* were present in over 50% of the surveyed GBR sponge species and were particularly abundant in *Cinachyra* sp., *X. exigua*, and *C. matthewsi*. Another recent investigation compared the presence of *Chloroflexi* in sponge species containing either high (HMA) or low (LMA) microbial abundance (Schmitt et al., 2011). Clear differences were observed between HMA and LMA species including a greater abundance, diversity and sponge-specificity of *Chloroflexi* in HMA sponges. In contrast, LMA species tended to be highly variable and contain *Chloroflexi* sequences related to seawater-derived microorganisms. These findings indicate a functional importance for *Chloroflexi* in HMA sponges and whilst we would predict that *Cinachyra* sp., *X. exigua*, and *C. matthewsi* from the GBR are HMA species based on sequence analysis of *Chloroflexi*, further microscopy analysis would be required to determine the microbial abundance within each of our GBR sponges.

No relationship between host phylogeny and bacterial communities were observed within the Dictyoceratida, Haplosclerida, Poescilosclerida, Halichondrida, and Pirophorida from the GBR. This finding is consistent with a 454 amplicon pyrosequencing study that surveyed sponge microbes across 32 different host species encompassing nine sponge orders (Schmitt et al., 2012b). Species within the same orders did not contain more similar microbial communities than species in different orders nor was there any clear correlation to host phylogeny when three species each within the genera *Aplysina, Hyrtios*, and *Ircinia* were compared (Schmitt et al., 2012b). Cospeciation of sponges and their symbionts would be most likely to occur if the microbial associates were transmitted strictly vertically (from adult to gametes/larvae). Research over the past decade has revealed that many sponges use both vertical (Usher et al., 2001; Oren et al., 2005; Enticknap et al., 2006; Schmitt et al., 2007; Sharp et al., 2007) and horizontal (Taylor et al., 2007; Schmitt et al., 2008; Webster et al., 2010) transmission strategies to maintain their complex and diverse microbial communities. The lack of correlation between host phylogeny and bacterial composition in the GBR sponges also suggests a strategy of symbiont acquisition that incorporates both vertical and horizontal transmission.

It has been hypothesized that abundant microbes common to all samples within a given habitat must be essential for the function of that community (Shade and Handelsman, 2012). Hence, the identification of core microbiomes (microorganisms that are shared between two or more samples) in complex microbial habitats can enhance our understanding of systems ecology. Across the 13 GBR sponge species investigated here, the core microbiome within each species was high yet the microbiome shared between species was low. However, it should be noted that additional sequencing for each species may reveal further sequences common to multiple samples. No OTUs were present in more than five different sponge species and 91% of the total OTUs were species-specific. Schmitt and colleagues (2012b) also identified a minimal core bacterial community in 32 different sponge species. In another study of five Mediterranean sponge species, 72% of the detected OTUs were species-specific, 26% were common to two or more species and only 2% were shared amongst all five species (Schmitt et al., 2012a). The study of three sympatric Mediterranean *Ircinia* sp. also revealed host species-specific assemblages and identified one proportion of the OTUs that were shared between the two most phylogenetically related species and a second component that was shared between the two species sharing the same cryptic habitat (Erwin et al., 2012a). These findings indicate that factors relevant to each host species can contribute to structuring the distinct microbial communities. Whilst next generation sequencing would further elucidate the host-specific nature of GBR sponge microbial communities, our results contribute to this growing body of evidence for species-specific microbial associations in sponges.

The vast majority of GBR sponges surveyed in the current study had high proportions of sequences that were more similar to other sponge-derived sequences than they were to sequences from the environment or other sources. These findings are consistent with our understanding of sponge-specific SC (Hentschel et al., 2002; Taylor et al., 2007; Simister et al., 2012a) and further our knowledge of their distribution and geographic range. In the global sponge microbial survey, each of the 32 species was also found to host bacteria that were more closely related to each other than they were to non-sponge derived bacterial species (Schmitt et al., 2012b). However, a recent survey of the rare microbial biosphere from ~650 marine samples collected from diverse habitats detected 77 of the 173 known sponge-specific SC in environmental (non-sponge) samples, although these were generally found at extremely low abundances (Taylor et al., 2012). To date, little is known about the functional roles of these “sponge-specific” bacteria although single celled genomics of a sponge-specific *Poribacteria* indicated potential symbiotic functions including autotrophic carbon fixation and vitamin B12 production (Siegl et al., 2011). Interestingly, sponge species from the present study which contained no sequences within sponge-specific clusters still maintained highly conserved bacterial populations, indicating that species without these “sponge-specific” sequences are still capable of structuring their communities and are not just reflecting the microbial composition of the surrounding seawater. For example, *Hamigera* sp. had no sequences within SC/SCCs and only 2% of *Paramyxilla* sp. fell within a SC yet the replicate individuals within each species shared 90 and 80% similarity respectively. Similarly, the highest number of sequences falling within SC/SCCs occurred in *L. variabilis* (92% of total sequences), yet this species had the lowest intra-species similarity based on microbial profiling (65%).

Whilst the number of microbes shared between different GBR sponge species was small, the core microbiome within each species was large, with the vast majority of microbes conserved in all replicate individuals per species. This indicates that these microbes are likely to be functionally important to the ecology of each species. Metagenomic sequence analysis recently highlighted how core symbiont functions can be provided in different sponge species by functionally equivalent microbes and analogous enzymes or biosynthetic pathways (Fan et al., 2012). Therefore, a “core microbiome” at the phylogenetic level does not need to exist across species. As indicated by Shade and Handelsman (2012), identifying the “core” microorganisms in any habitat is essential for defining the healthy community and subsequently predicting how the community will respond to perturbation. Given the increasing incidence of sponge disease and health declines associated with climate change, an enhanced understanding of the species-specific symbionts in GBR sponges will be a valuable resource.

### Conflict of interest statement

The authors declare that the research was conducted in the absence of any commercial or financial relationships that could be construed as a potential conflict of interest.
